# Visualization and quantification of the degenerative pattern of the distal tibia and fibula in unilateral varus ankle osteoarthritis

**DOI:** 10.1038/s41598-021-00874-7

**Published:** 2021-11-03

**Authors:** Hiroyuki Seki, Naomichi Ogihara, Tetsuro Kokubo, Takeo Nagura

**Affiliations:** 1 Department of Clinical Biomechanics, Keio University, 35 Shinano-machi, Shinjuku-ku, Tokyo, 160-8582 Japan; 2 Department of Orthopaedic Surgery, Ogikubo Hospital, 3-1-24 Imagawa, Suginami-ku, Tokyo, 167-0035 Japan; 3 Department of Orthopaedic Surgery, International University of Health and Welfare Mita Hospital, 1-4-3 Mita, Minato-ku, Tokyo, 108-8329 Japan; 4 Department of Biological Sciences, Graduate School of Science, The University of Tokyo, 7-3-1 Hongo, Bunkyo-ku, Tokyo, 113-0033 Japan; 5 Department of Orthopaedic Surgery, Tachikawa Hospital, 4-2-22 Nishiki-cho, Tachikawa-shi, Tokyo, 190-8531 Japan

**Keywords:** Bone, Diseases

## Abstract

The present study aimed to quantify and visualize the degenerative patterns of the distal tibia and fibula due to ankle osteoarthritis (OA). We analyzed differences in tibial and fibular surface deviation between sides of patients with unilateral varus ankle OA (medial talar tilt > 4°) by registering each surface model to the mirror image of corresponding bone. Computed tomography images of both feet of 33 patients (OA: 22, control: 11) were examined. Statistically significant surface depression of approximately 2.5 mm on the anterior articular surface of the medial malleolus, and surface elevation of approximately 1 mm on the anterodistal edge of the tibiofibular joint and the lateral malleolus were observed in OA patients. These bone degenerations were found to be correlated with those on the other side of the ankle joint, the medial margin of the talar trochlea and the lateral articular surface of the talus, respectively. In contrast, the amount of bone depression on the plafond was smaller than previously anticipated. Such quantitative information about stereotypical patterns of bone degeneration in ankle OA would contribute to better understanding of the development of ankle OA and possible therapeutic interventions.

## Introduction

Ankle osteoarthritis (OA) is a common progressive disease characterized by destruction of articular cartilage and bony degeneration around the tibiotalar joint^1^. In addition, coronal-plane varus malalignment at the level of the tibiotalar joint is often observed in ankle OA^2^. Previous studies have examined the morphological characteristics of the distal tibia in varus ankle OA using plain radiographs and reported that varus tilt of the tibia, anterior opening of the plafond, and distal opening of the articular surface of the medial malleolus^3–5^ were the main morphological manifestations in ankle OA. However, two-dimensional assessment based on a plain radiograph is not sufficient to capture the complex morphology of osteophyte formation and bone resorption in ankle OA, which is essentially three-dimensional.

A few published studies have assessed the three-dimensional morphology of the ankle mortise in ankle OA using computed tomography (CT). Wiewiorski et al.^6^ showed that the anteroposterior width and sagittal curvature of the distal tibia were significantly larger in ankle OA than in the normal ankle. Similarly, Schaefer et al.^7^ reported that larger radii of the tibial plafond were significantly correlated with the severity of ankle OA. However, to the best of authors' knowledge, those are the only studies published so far. Detailed assessment and quantification of the pattern of the morphological degeneration of the ankle joint formed by the tibia, fibula, and talus have not been previously attempted due to difficulties associated with the large inter-individual variations in the size and shape of these ankle bones. Therefore, quantitative information about degenerative patterns of osteophyte formation or bone resorption of the ankle bones due to ankle OA has been largely unexplored.

Given this situation, we recently proposed a method to quantify and visualize stereotypical patterns of bone degeneration occurring in OA tali^8^. In that study, we suggested that left–right comparison of ankle bones in patients with unilateral ankle OA allows quantification and visualization of the patterns of bone degeneration occurring in ankle OA because the effect of the large inter-individual variabilities in the bone morphology can be eliminated, since left and right tali of healthy human are basically symmetrical^9^. By applying this method, the study successfully visualized and quantified the stereotypical patterns of degeneration occurring in OA tali. However, in our previous study, the degenerative patterns of the distal tibias and fibulas in ankle OA were not investigated.

In the present study, therefore, the aim was to visualize and quantify three-dimensionally the patterns of morphological degeneration of the distal tibia and fibula in patients with varus ankle OA by the left–right comparison of the bones in patients with unilateral ankle OA. Specifically, we tested if the left–right surface deviations of the tibia and fibula in unilateral varus ankle OA patients are significantly larger than those of healthy humans. It has been reported that there is a slight left–right asymmetry in the human tibia and fibula^10^, but if the surface deviations in the unilateral varus ankle OA patients are significantly larger than the surface deviations due to this inherent asymmetry in healthy humans, we can firmly conclude that the extracted surface deviations in the patients are due to degeneration in ankle OA.

## Results

Based on the plain radiographs, 22 patients in the OA group were found to be in stage 3a, 3b, or 4 on the OA side and in stage 1 or less on the opposite non-OA side, according to the Takakura classification. The numbers of patients classified into each stage were five, eleven, and six, respectively. Eleven patients in the control group were not diagnosed as having ankle OA based on the CT findings. There were no significant differences between the two groups in age and the sex ratio.

**Figure 1:**
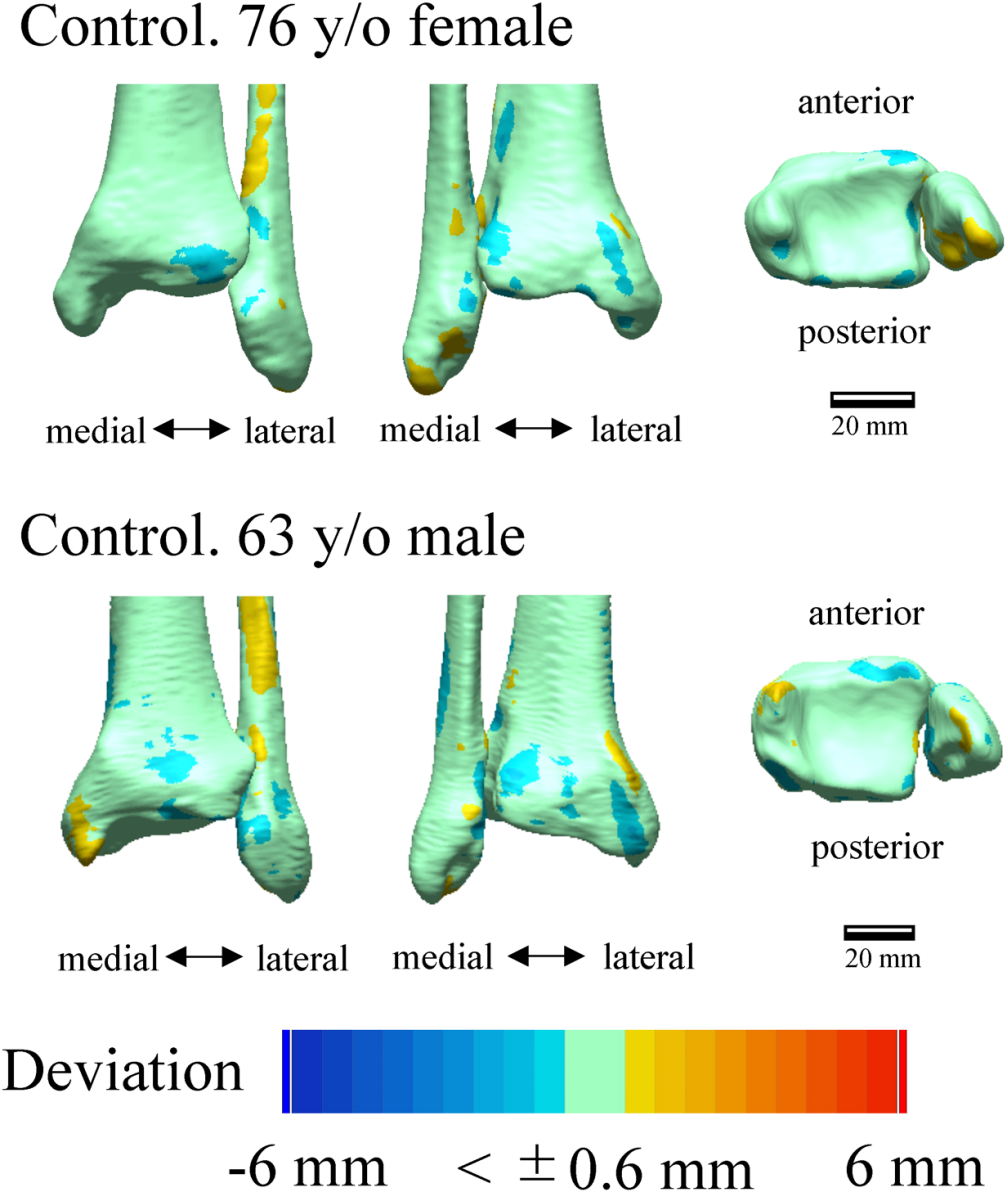
Deviation color maps of left–right comparisons of the tibia and fibula in the control group. Two representative examples are presented. The red and blue colors are deviations of the right surface outside and inside of the left surface. 1. Anterior view. 2. Posterior view. 3. Inferior view. y/o = years old.

**Figure 2:**
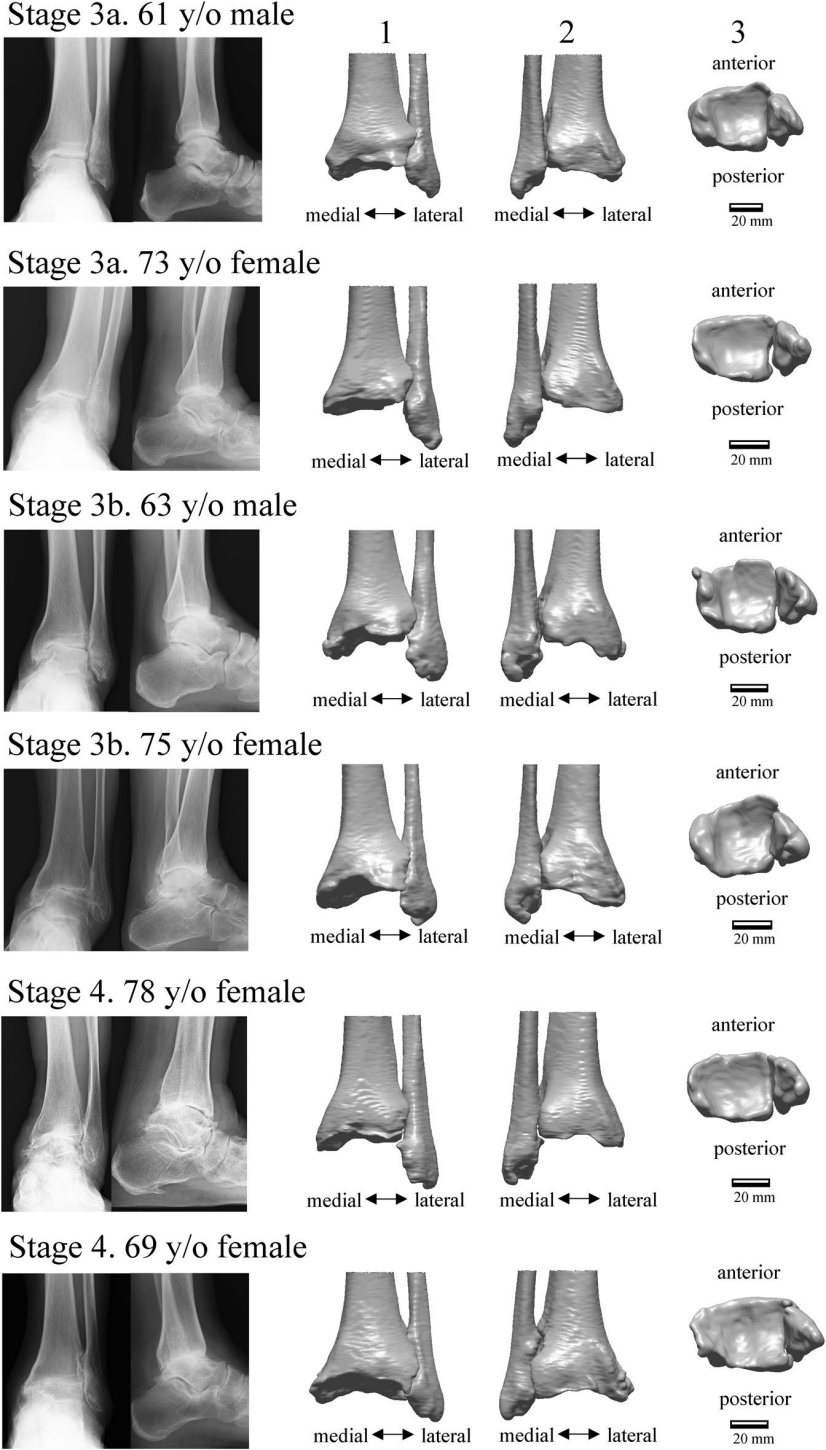
Plain radiographs and bone surface models reconstructed based on CT data of six representative cases, two for each stage, in the osteoarthritis (OA) group. 1. Anterior view. 2. Posterior view. 3. Inferior view. y/o = years old.

**Figure 3:**
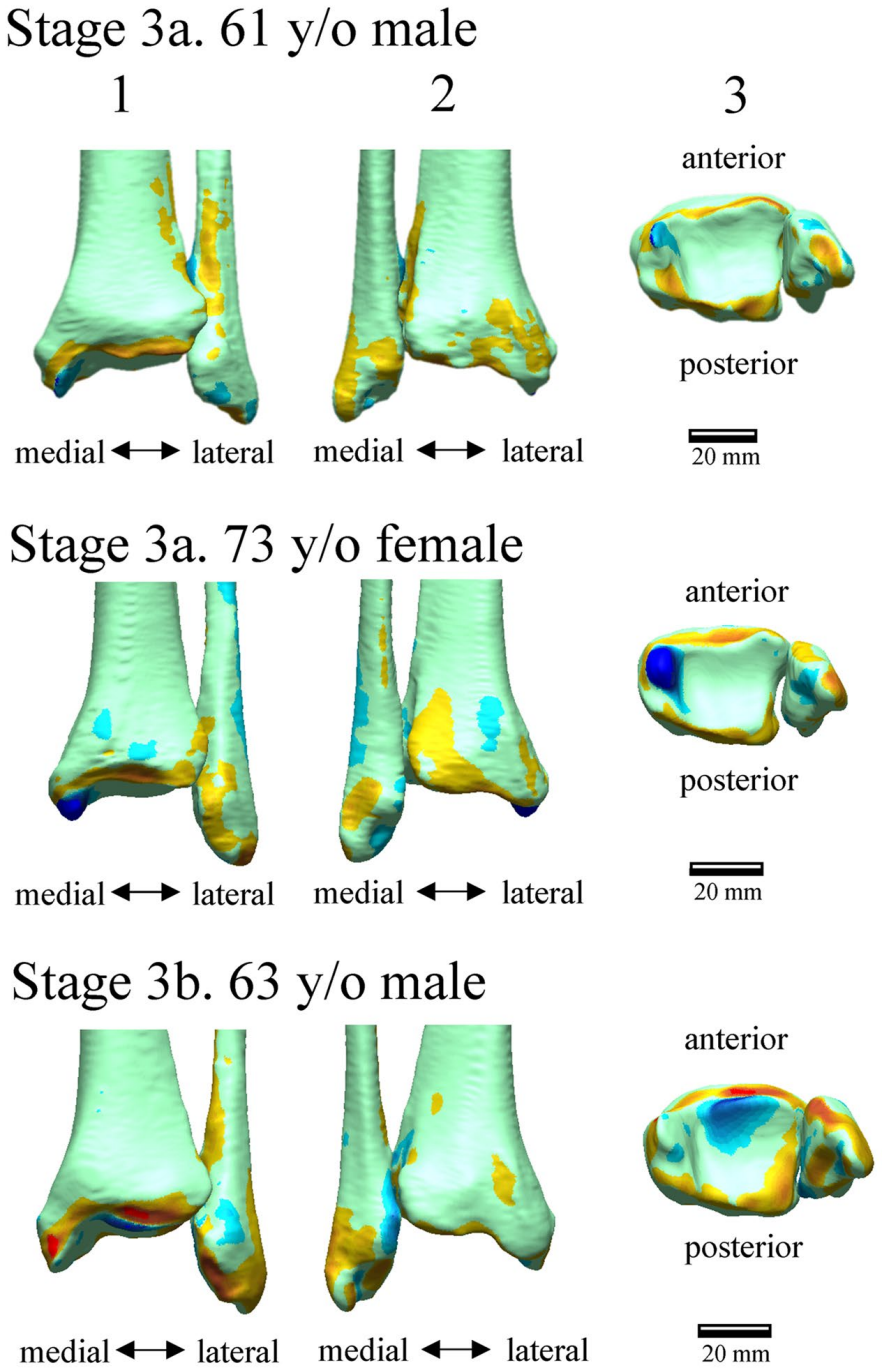

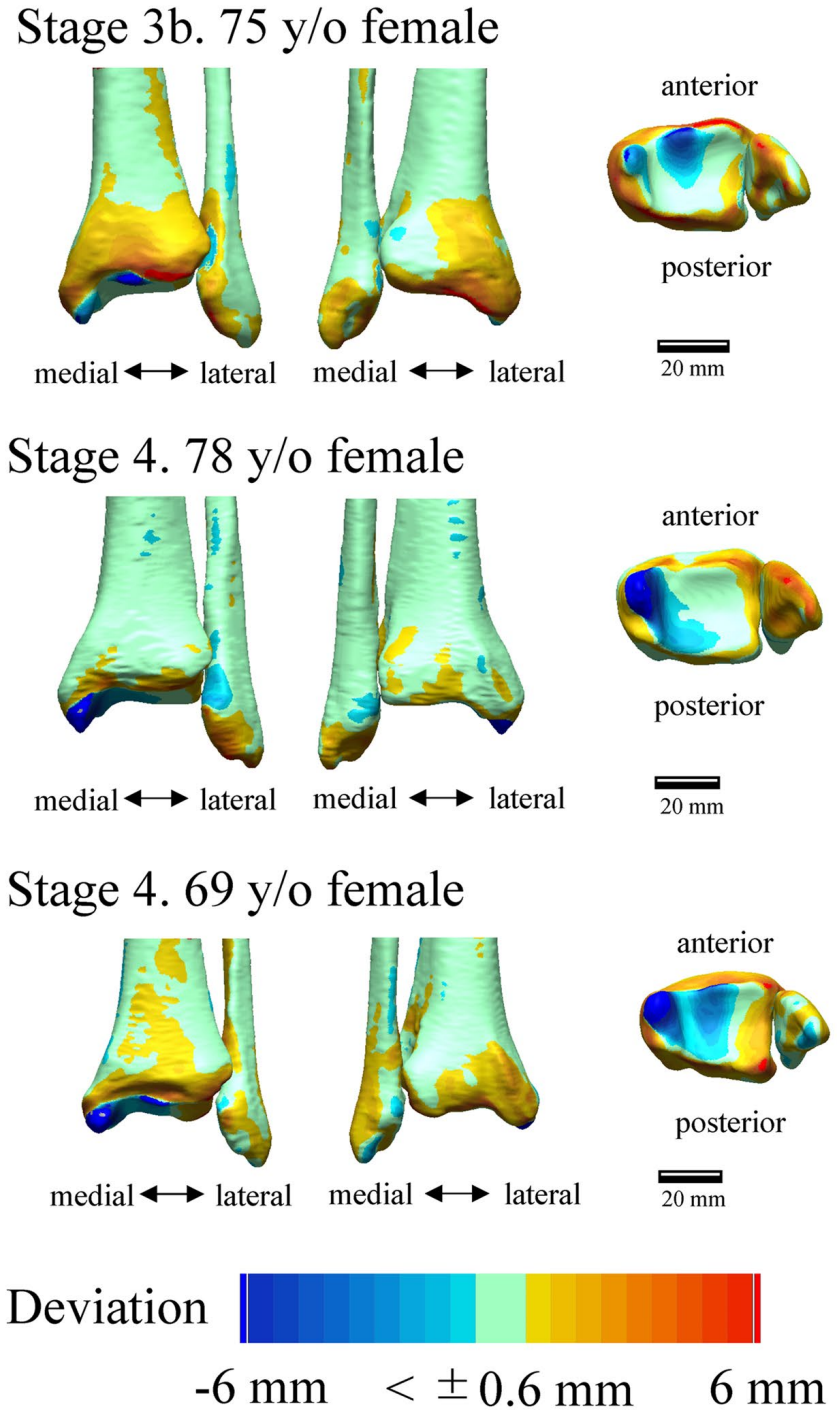
Deviation color maps of left–right comparisons of the tibia and fibula in the OA group. Six representative cases (the same cases as in Fig. 1) are presented. The red and blue colors are deviations of the OA surface outside and inside of the opposite surface. 1. Anterior view. 2. Posterior view. 3. Inferior view. y/o = years old. See text for more details.

Figure 1 shows the deviation color maps of two exemplary subjects within the control group. The left–right surface differences of the distal tibia and fibula were very small in the control group. Figure 2 shows the weight-bearing plain radiographs and bone surface models reconstructed based on CT data of the OA-side in six representative cases. The deviation color map of almost all cases of all stages showed surface depression on the medial articular surface of the medial malleolus and surface elevation along the anterolateral margin of the distal tibia (Fig. 3). In stage 3b, some surface depression on the middle of the anterior area of the plafond was observed. However, the change in the surface of the medial and posterior areas of the plafond was found to be small (< 0.6 mm) compared with that of the articular surface of the medial malleolus (See below for quantitative comparisons). In addition, surface elevation on the anterior borders of the lateral malleolus and the distal tibiofibular articular surface was found.

**Figure 4:**
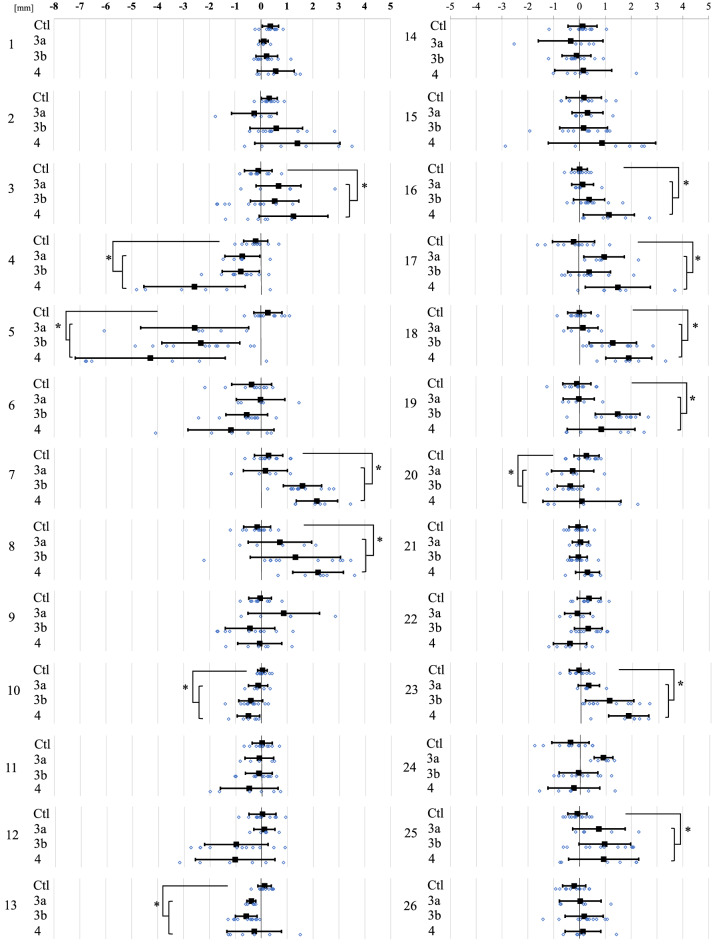
The amounts of left–right surface deviations in the OA and control groups at 26 regions of interest (See Table 1 and Fig. 5). Error bars indicate standard deviations. The deviations are positive if the surface of the OA or right surface is outside the opposite non-OA or left surface, and negative if the surface is inside. Three OA groups (3a, 3b, and 4) were pooled for statistical comparisons with the corresponding control. *Significant difference between the pooled OA and the control, *p* < 0.05.

**Figure 5:**
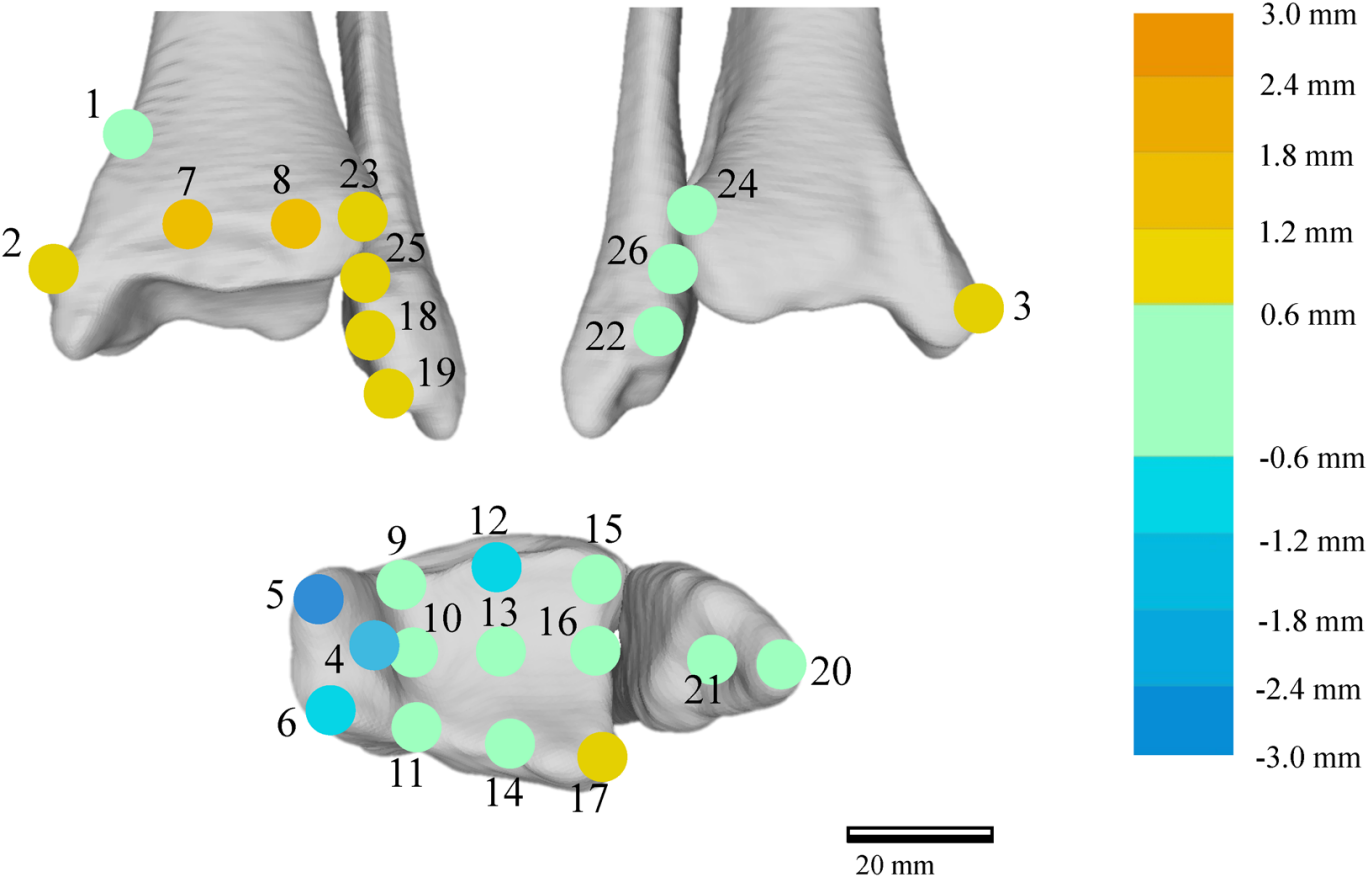
The color map of the mean surface deviation in the OA group at the 26 regions of interest (See Table 1).

Figure 4 shows the amounts of surface deviation in the OA and control groups at 26 regions defined on the distal tibia and fibula surface (See “Methods” section and Table 1). Three OA groups (3a, 3b, and 4) were pooled for statistical comparisons with the corresponding control. The amount of surface depression on the medial malleolus (regions 4 and 5) was the largest, with a mean deviation of more than 2.5 mm in severe varus ankle OA (Fig. 5). Although the surface elevations on the anterior apophysis of the distal tibia (regions 7 and 8) were large, with a mean deviation of more than 2 mm in severe varus ankle OA, the surface depression of the plafond except for the anterior area was relatively small, with mean deviation of less than 0.6 mm (regions 9–11, and 13–16) (Fig. 5). The mean amounts of surface elevation on the anterior borders of the lateral malleolus and the distal tibiofibular joint were approximately 1 to 2 mm in varus ankle OA with stage 3b and 4 (regions 18, 19, 23, and 25) (Figs. 4, 5). On the other hand, the mean left–right surface differences in the control group were < 0.4 mm, indicating the above-mentioned surface deviations occurred due to varus ankle OA.

**Table 1:** The 26 landmarks used to define the regions of interest.

	Definitions
1	Midpoint of the medial concavity of the medial malleolus
2	Most anterior point on the medial apophysis of the medial malleolus
3	Most posterior point on the medial apophysis of the medial malleolus
4	Centroid of the articular surface of the medial malleolus
5	Most inferior point on the anterior colliculus of the medial malleolus
6	Most inferior point on the posterior colliculus of the medial malleolus
7	Most medial point on the anterior apophysis of the distal tibia
8	Most lateral point on the anterior apophysis of the distal tibia
9	Most anterior point on the medial margin of the plafond
10	Midpoint on the medial margin of the plafond
11	Most posterior point on the medial margin of the plafond
12	Midpoint of the anterior margin of the plafond
13	Centroid of the plafond
14	Midpoint of the posterior margin of the plafond
15	Most anterior point on the lateral margin of the plafond
16	Midpoint on the lateral margin of the plafond
17	Most posterior point on the lateral margin of the plafond
18	Midpoint on the anterior border of the lateral malleolus
19	Most inferior point on the anterior border of the lateral malleolus
20	Tip of the lateral malleolus
21	Centroid of the articular surface of the lateral malleolus
22	Most posterior point on the digital fossa of the lateral malleolus
23	Most anterior point on the distal tibiofibular articular surface of the tibia
24	Most posterior point on the distal tibiofibular articular surface of the tibia
25	Most anterior point on the distal tibiofibular articular surface of the fibula
26	Most posterior point on the distal tibiofibular articular surface of the fibula

Table 2 shows the correlations of the surface deviations on the tibia and fibula with the corresponding surface deviations on the talus (See “Methods” section). It was found that the surface depressions on the articular surface of the medial malleolus (regions 4 and 5) were significantly correlated with the surface depressions on the medial margin of the talar trochlea (regions 2 in Seki et al.^8^). Furthermore, there was a significant correlation between the surface elevations on the anterior apophysis of the distal tibia (regions 7 and 8) and the anterior margin of the talar trochlea (region 4 in Seki et al.^8^). In addition, the surface elevations on the anterior edge and articular surface of the lateral malleolus (region 18, 19, and 21) and the anterior edge of the distal tibiofibular articular surface (region 23 and 25) were significantly correlated with the surface elevation on the lateral articular surface of the talus (region 11 in Seki et al.^8^).

**Table 2:** Correlations of the left–right surface deviations between the distal tibia and fibula and the talus.

Area	Region of distal tibia and fibula	Region of talus	*rs*	*p*-value
Medial	4	2	0.591	< 0.001
	5	2	0.779	< 0.001
	5	3	0.414	0.019
Anterior	7	4	0.505	0.004
	8	4	0.432	0.014
Lateral	18	11	0.539	0.002
	19	11	0.433	0.014
	23	11	0.549	0.001
	25	7	0.405	0.022
	25	11	0.396	0.026

Results were presented if the correlations were statistically significant (*p* < 0.05). See Table 1 and Fig. 5 for the regions of the distal tibia and fibula. The regions of the talus were defined in our previous study^8^: region 2, the most superior point of the medial margin of the trochlea; region 3, the most posterior point of the medial margin of the trochlea; region 4, the midpoint of the anterior margin of the trochlea; region 7, the most anterior point on the lateral margin of the trochlea; region 11, the most inferior point of the lateral articular facet. *rs*, Spearman’s rank correlation coefficient.

## Discussion

Previous studies have compared the morphological characteristics of the distal tibia and fibula between patients with varus ankle OA and healthy subjects by approximating the articular surface with a line on plain radiographs to quantify the inclination angles of lines^3–5^ or with a circle on CT images to calculate the radii of circles^6,7^. However, the extracted differences in these studies were not confined to true degeneration of the bones due to ankle OA, but they included possible morphological differences due to congenital inter-individual variations in size and shape of the bones. In addition, these methods approximating the articular surface with a line or a circle are too crude to express complex, uneven patterns of bony degeneration, such as osteophytes and surface depressions observed in ankle OA. In the present study, surface deviations of the left and right ankle bones in patients with varus ankle OA^8^ were quantified three-dimensionally to successfully extract and separate genuine patterns of morphological degeneration of the distal tibia and fibula.

The present study demonstrated that there exists a characteristic pattern of bone degeneration of the distal tibia and fibula in varus ankle OA, which has not been previously described based on plain radiographs. Specifically, surface depressions on the anterocentral area of the articular surface of the medial malleolus and surface elevations on the anterior edge of the lateral malleolus were observed in varus ankle OA, but such bone degeneration was not observed in the control group. Although these bony degenerations have been empirically observed during surgery, this study offered, for the first time, quantitative evidence showing that such stereotypical patterns of bone degeneration actually occurring in the distal tibia and fibula in patients with varus ankle OA. Understanding the pattern of the bone degeneration in ankle OA may contribute to clarifying the mechanism underlying the abnormality of ankle joint kinematics in ankle OA.

In the present study, the largest bone degeneration (surface depression) was found to occur on the articular surface of the medial malleolus in varus ankle OA. In addition, this was found to be correlated with the bone degeneration of the medial margin of the talar trochlea. This fact possibly indicates that large repetitive stress had been concentratively applied to this region in patients with varus ankle OA causing deterioration and erosion of the joint surface and development of OA^11^. If the joint erosion happened, the corresponding joint space width would increase and the stability of the talar mortise would decrease. Axial loading of the human foot is known to result in eversion of the calcaneus and inertial rotation of the talus and tibia due to innate mobility of the human foot so-called tibio-calcaneal coupling^12–14^. If the joint space width of the ankle mortise got larger, the talus would rotate larger in the direction of internal rotation. Several studies using weight-bearing CT have reported that the abnormal internal rotation of the talus in the axial plane was a characteristic pathological feature of varus ankle OA^15,16^. The more internally rotated posture of the talus in varus ankle OA was possibly due to the characteristic pattern of surface depression of the articular surface of the medial malleolus.

The present color map analysis also showed that surface depressions in the anterocentral area of the plafond occurred in varus ankle OA. This is consistent with the previous report that the angle between the tibial shaft and the distal joint surface (plafond) on the sagittal plane (i.e., anterior opening of the ankle joint) was larger^4^ and the plafond was more flattened^6,7^ in patients with ankle OA, because if the anterior surface of the arch-shaped plafond was depressed, the sagittal curvature of the plafond would be reduced. The present study also observed that surface elevations were produced on the anterior apophysis of the distal tibia, along with the surface elevation on the anterior area of the talar trochlea in ankle OA. In patients with varus ankle OA, the talus is reportedly subluxated anteriorly by anterior shear force induced by dysfunction of the anterior talofibular ligament^3,17^. These surface elevations, which may be attributed to osteophyte formations on the anterior apophysis of the distal tibia and on the anterior surface of the talar trochlea are likely produced by accumulation of large loading stress acting on the anterocentral talocrural articular surface as a result of the subluxation of the talus in varus ankle OA^18^. It must be noted, however, that this osteophyte formation, if marginal, may contribute to stabilizing the ankle joint with antero-posterior hypermobility^19,20^.

The present study also demonstrated surface elevations on the anterior borders of the lateral malleolus and the distal tibiofibular articular surface in ankle OA. This deviation corroborates our previous study showing that surface elevations were also produced on the lateral articular surface of the talus^8^. Larger loading stress is possibly accumulated to the lateral side, but not the medial side of the trochlea in ankle OA during standing and walking, possibly to reduce pain due to damage to cartilage and subchondral bone on the anteromedial side of the talar trochlea^8^. The surface elevations might also be associated with abnormal distal tibiofibular joint mobility in ankle OA. A previous CT study proposed that the fibula was more externally rotated in severe ankle OA^21^, possibly due to instability of the talus in the ankle mortise in the axial plane. The fibula rotates externally and the anterior width of joint space is widened during external rotation of the talus at the distal tibiofibular syndesmosis^22,23^. The overload of the syndesmosis due to excessive internal–external mobility may cause the osteophyte formation on the anterior borders of the distal tibiofibular joint. This should be confirmed in future research by comparative evaluation of the position of the fibula with respect to the tibia in ankle OA using weight-bearing CT^24^.

This analysis has some limitations. The method in this study can be applied only to patients with unilateral ankle OA, not to patients with bilateral ankle OA, since comparison with the non-OA side was performed for quantification of degeneration of the distal tibia and fibula. However, this is indispensable to visualize and quantify the pattern of morphological degeneration of the distal tibia and fibula while eliminating the effects of the large inter-individual variabilities of bone morphology. Secondly, the bone model reconstructed from CT scan should ideally have a better accuracy. However, the resolution of the CT scan in the present study is nearly the highest we can get if both feet are scanned at one time to minimize doses of x-ray using a conventional medical CT scanner. In addition, the extracted differences in the bone degeneration were generally > 1 mm, much larger than the accuracy of the bone model in the present study. Therefore, this limitation should not have a major effect on the current results. Thirdly, the subjects in this study include more females than males, although there was no significant difference in the ratio of male to female between the control and the OA groups. Several studies have reported sex-related morphological variations in the tibia and talus^25,26^. Imbalance in the number of female and male participants may have influenced the outcome of this study. However, we believe that such effect of gender should be minor. Lastly, the present study does not answer to the question of how the observed bone degeneration in varus ankle OA was developed. There are several processes of bone deposition and remodeling involved in joint pathophysiology; the observed surface depression could be due to factors such as bone resorption, impact, and erosion of bone surface^27,28^, but the present study does not answer which factor is more responsible than the others. To trace the pathogenetic process and mechanism underlying the observed bone surface depression and elevation is difficult but should be investigated in future studies.

## Conclusion

In conclusion, the morphological bony degeneration of the distal tibia and fibula due to varus ankle OA was characterized visually as surface depression on the anterior area of the medial malleolus articular surface and surface elevation on the anterior apophysis of the distal tibia, the anterior margin of the lateral malleolus, and the anterior margin of the distal tibiofibular joint. This morphological information is possibly useful for better understanding of the development of varus ankle OA.

## Methods

### Patient selection

The present research was approved by the Institutional Review Board of Tachikawa hospital (Tokyo, Japan), and informed consent was obtained from all subjects. The methods were performed in accordance with the approved guidelines. A total of 33 patients (average age, 69.5 years; range, 49 to 88 years; 7 males, 26 females), consisting of an ankle OA group diagnosed with unilateral varus ankle OA (22 patients; 71.1 years; 49 to 88 years; 5 males, 17 females) and a control group (11 patients; 66.2 years; 54 to 78 years; 2 males, 9 females), were included. Varus ankle OA was diagnosed based on a measured^15^ talar medial tilt angle > 4° and stage ≥ 3a according to the classification reported by Tanaka et al.^3^ on weight-bearing anterior–posterior plain radiographs. Subjects with ankle OA following fractures of the tibia, fibula, or talus or inflammatory diseases such as rheumatoid and infectious arthritis were excluded. Both secondary ankle OA subjects following repetitive ligamentous injuries and primary ankle OA subjects with unknown sprain histories were included in this study. The classification of the ankle OA group was independently diagnosed by two of the authors, who are foot and ankle orthopedic surgeons with more than 10 years of experience (H.S. and T.K.), and the classification results were completely consistent with each other. The control group included subjects with fresh trauma without involvement of the tibia and fibula, such as fractures of the talus, calcaneus, or forefoot. The diagnostic process with interviews confirmed that all patients were free of any foot and ankle pathologies except for the above fresh trauma.

### Bone model reconstruction

**Figure 6:**
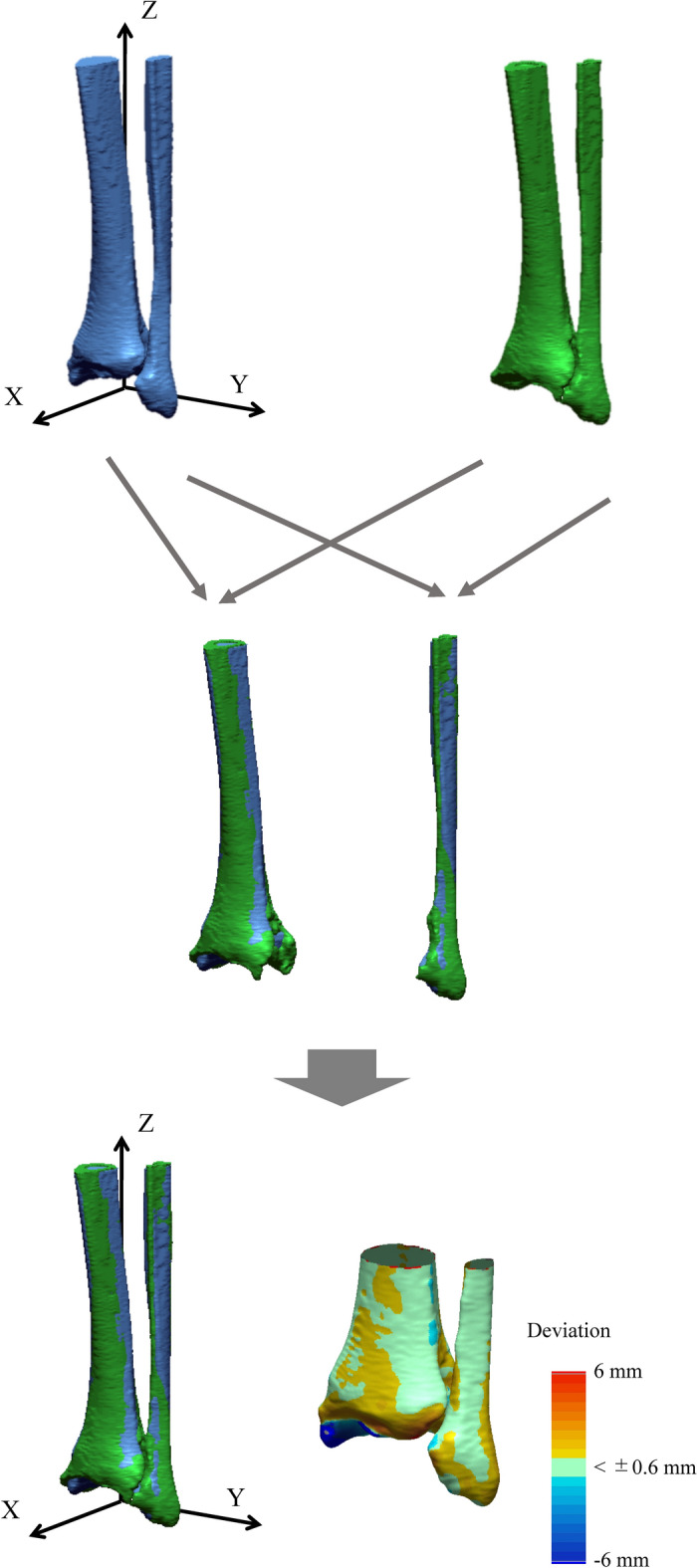
Left–right comparisons of the tibia and fibula in unilateral varus ankle OA. Mirror image models are created for the right-side specimens, and the osteoarthritic tibia and fibula models (green) are respectively registered to the opposite non-OA tibia and fibula models (blue) via an iterative closest point algorithm. The surface deviation is calculated as the distance between the two surfaces along the surface normal direction of the non-OA surface. The red and blue colors are deviations of the osteoarthritic surface outside and inside of the non-OA surface.

CT scan data of the right and left hindfeet of all subjects were obtained using a CT scanner (Aquilion 64, Toshiba Medical Systems Corporation, Otawara, Japan, or SOMATOM Definition AS 64, Simens Healthineers, Tokyo, Japan). The pixel size of the CT images ranged from 0.52 to 0.65 mm, and the reconstructed slice thickness was 0.5 mm; therefore, the voxel was anisotropic. The left and right tibia and fibula were then each reconstructed in a virtual three-dimensional space using specialized software (Avizo 9.1; FEI Visualization Sciences Group, Hillsboro, OR, USA). The accuracy of the three-dimensional bone surface model reconstructed from CT scan was reportedly about half to one pixel size (i.e., approximately 0.3 to 0.6 mm)^29–31^. Mirror image models were created for the right-side models, so that all models could be treated as left-side models. To facilitate visual comparisons, a body-fixed coordinate system of the tibia and fibula complex (the x-, y- and z-axes representing the anteroposterior, mediolateral, and dorsoplantar axes, respectively) was defined by the modified method for the coordinate system recommended by the International Society of Biomechanics^32,33^ (Fig. 6).

### Surface deviation analysis

To extract possible left–right differences in the tibial and fibular morphologies of each participant, the OA tibial and fibular models were each registered to the opposite non-OA models by an iterative closest point (ICP) algorithm^34^ (Fig. 6), using RapidForm OR3 64 Hotfix3.1 (Inus Technology, Seoul, Korea). The ICP algorithm iteratively transforms the OA model to match the non-OA model so as to minimize the distance between the two sets of vertices constituting the two models. The surface deviation was calculated as the distance between the two surfaces along the surface normal direction of the non-OA model, with the light green color indicating that the deviation was less than 0.6 mm, and the red and blue colors indicating deviations of the OA model outside and inside of the non-OA model.

To statistically evaluate the differences in the surface deviation of tibial and fibular morphologies, 26 regions of interest on the surfaces of the non-OA ankles (left ankles for the control group) were defined, and mean deviations were calculated (Fig. 5). The 26 regions consisted of 6 regions on the medial malleolus (#1–6), 11 regions on the plafond (#7–17), 5 regions of the fibula (#18–22), and 4 regions on the distal tibiofibular joint (#23–26). Each region of interest was defined as the surface area within a radius of 1 mm from the corresponding landmark digitized on the model (Table 1). For each vertex on the OA or right model within the sphere, the closest distance to the surface of the non-OA or left model was calculated^35^. The sign of the distance was determined based on the direction of the vertex normal vector (the average of the combined facet normal vectors of all connected polygons using the surface area of each face as the weight). The mean deviations were calculated for each region to quantify and statistically evaluate the degrees of surface elevation or depression observed on the OA tibia and fibula with respect to the non-OA tibia and fibula (on the right with respect to the left in the control group). Additionally, the color maps of the mean deviations were provided for the control (Fig. 1) and each of the three stages (Fig. 3).

To investigate possible correlation in the pattern of bone degeneration between the tibia and fibula, and the talus, the calculated surface deviations in the present study were compared with our previous study reporting the surface deviations on the talus in the same varus ankle OA patients^8^. Specifically, nine regions where the magnitudes of the surface deviations were > 0.6 mm and significantly different between the pooled OA and control groups were selected (regions 4, 5, 7, 8, 17–19, 23 and 25). We then assessed the correlations of the surface deviations of the regions with the corresponding surface deviations on the other side of the ankle joint reported in our previous study^8^. However, the control group in our talus study was different from that of the present study. Therefore, the surface deviations on the talus in the control group in the present study were newly obtained as described previously.

### Statistics

Pearson’s chi-squared test was used to compare the ratio of gender between the control group and the OA group. The Wilcoxon test was used to assess the left–right surface deviations of the control group from zero. The Mann–Whitney U test were used to compare the ages and the surface deviations between the control and OA groups. The Spearman's rank correlation was used to analyze the relationship between the surface deviations of the tibia and fibula and those of the talus. The level of significance was *p* < 0.05. Statistical analyses were performed using EZR (Saitama Medical Center, Jichi Medical University, Saitama, Japan), which is a graphical user interface for R (The R Foundation for Statistical Computing, Vienna, Austria). More precisely, it is a modified version of R commander designed to add statistical functions frequently used in biostatistics^36^.

## Data Availability

The authors declare no restrictions on the availability of material and data to the publishing team.
